# Embryo survival in the oviduct not significantly influenced by major histocompatibility complex social signaling in the horse

**DOI:** 10.1038/s41598-020-58056-w

**Published:** 2020-01-23

**Authors:** E. Jeannerat, E. Marti, S. Thomas, C. Herrera, H. Sieme, C. Wedekind, D. Burger

**Affiliations:** 1 Swiss Institute of Equine Medicine ISME, University of Berne, and Agroscope, Avenches, Switzerland; 2 Department of Clinical Research, Vetsuisse Faculty, University of Berne, Berne, Switzerland; 3 Clinic for Animal Reproduction Medicine, Vetsuisse Faculty, University of Zurich, Zurich, Switzerland; 4 Unit for Reproductive Medicine – Clinic for Horses, University of Veterinary Medicine Hannover, Hannover, Germany; 5 Department of Ecology and Evolution, Biophore, University of Lausanne, Lausanne, Switzerland

**Keywords:** Ecology, Behavioural ecology

## Abstract

The major histocompatibility complex (MHC) influences sexual selection in various vertebrates. Recently, MHC-linked social signaling was also shown to influence female fertility in horses (*Equus caballus*) diagnosed 17 days after fertilization. However, it remained unclear at which stage the pregnancy was terminated. Here we test if MHC-linked cryptic female choice in horses happens during the first days of pregnancy, i.e., until shortly after embryonic entrance into the uterus and before fixation in the endometrium. We exposed estrous mares to one of several unrelated stallions, instrumentally inseminated them with semen of another stallion, and flushed the uterus 8 days later to test for the presence of embryos. In total 68 embryos could be collected from 97 experimental trials. This success rate of 70.1% was significantly different from the mean pregnancy rate of 45.7% observed 17 days after fertilization using the same experimental protocol but without embryo flushing. Embryo recovery rate was not significantly dependent on whether the mares had been socially exposed to an MHC-dissimilar or an MHC-similar stallion. These observations suggest that MHC-linked maternal strategies affect embryo survival mainly (or only) during the time of fixation in the uterus.

## Introduction

The major histocompatibility complex (MHC) plays a key role in the adaptive immune response of vertebrates^1^. MHC molecules present antigen peptides to T cells enabling the immune system to recognize pathogens^2^. The MHC also influences social signaling, as demonstrated in more than 20 species so far^1,3,4^. In humans, for example, at least 16 studies provide evidence for MHC-related odors or odor preference^5^, and further studies report evidence for MHC effects on mate choice or sexual responsivity in our species^6^. MHC-linked social signaling is also well established in the horse (*Equus caballus*)^7–10^.

MHC-linked social signaling may not only influence kin recognition and mate choice but also maternal investment into a pregnancy^1,3,4^, either to avoid male infanticide (“Bruce effect”^11,12^) or as a late form of sexual selection that favors certain MHC genotypes over others^13^. In the former case, MHC-linked signals that reveal the presence of a new dominant male can induce a pregnancy block^14,15^ while MHC matching between male and female would not be expected to matter^16^. In the latter case, i.e. if maternal investment into a given pregnancy reveals female preferences for certain male genotypes, higher rates of failed pregnancies would be predicted for MHC-similar than for MHC-dissimilar pairs^3,4^. Indeed, human couples that share MHC antigens frequently suffer from a higher prevalence of recurrent spontaneous abortions than couples that do not share MHC antigens^17,18^, and Ober *et al*.^19^ found reduced fecundity of MHC-similar couples within Hutterites, a group of people with usually large family sizes. These MHC effects seemed not linked to inbreeding^19^. Women (or females in general) are not expected to have conscious control over these physiological and biochemical processes.

Burger *et al*.^9^ used horses to test experimentally whether MHC social signaling can affect female fertility. They found higher rates of pregnancies after instrumental insemination when mares had been socially exposed to an MHC-dissimilar stallion around the time of fertilization than when they had been exposed to an MHC-similar stallion, while overall genetic relatedness between stallions and mares did not play a role. However, their study was performed in the context of a commercial breeding program, i.e. pregnancies could only be determined by transrectal ultrasonography 14–17 days after ovulation, and it remained unclear whether cryptic female choice happened in the oviduct or during fixation of the embryo in the endometrium. Here we use the horse again (and a new sample of mares than used by Burger *et al*.^9^) to test if MHC social signaling affects cryptic female choice during the first 8 days after ovulation, i.e. before fixation of the embryo in the endometrium would start.

The first 8 days of gestation include zygote formation, oviductal passage of the embryo, and its entrance into the uterus. In the horse, fertilization of one or rarely two oocytes occurs within 24 hours after ovulation in the ampulla of the oviduct^20^, followed by cell division and development to the morula and blastocyst stages^21,22^, and prostaglandin E_2_-mediated transport towards the isthmus^23^. Equine embryos then leave the oviduct around 6.5 days after ovulation^24^. After their entrance into the uterus, equine embryos move freely inside of the uterine lumen. This entails a suppression of prostaglandin F_2α_ release by the endometrium, which would otherwise result in luteolysis of the corpus luteum and early pregnancy failure^25,26^. This process, starting at around day 10, is traditionally defined as “maternal recognition of pregnancy”^27^, even if embryo-maternal signaling probably occurs also before this stage^28^. Equine embryos take a fixed position in the endometrium usually around day 16 (the timing of this event seems to range from day 13 to day 18^29,30^). Embryos then prepare their attachment, mediated by progesterone, until successful implantation in the endometrium at around day 40 of pregnancy^31^. First diagnosis of pregnancy in horses is typically performed around 14 to 17 days after ovulation by transrectal ultrasonography^32^.

Research on MHC-linked sexual selection has focused much on mate choice, while processes that are likely to happen after mating are not sufficiently understood yet. They may have important consequences for various evolutionary processes, including pathogen-host coevolution. Understanding MHC-linked female strategies during early gestation may also be important in reproductive medicine and in animal breeding. In the horse breeding industry, for example, fertility rates are typically lower than those observed under feral conditions, potentially due to a lack of social interactions between stallions and mares^33,34^. Allowing for MHC-linked social communication may increase reproductive efficiency not only in horses but in other farm animals, pets, zoo animals, or animals bred in the context of species conservation^35^.

## Results

The 29 mares could be tested for in total 97 cycles, corresponding to 1 to 7 cycles per mare. Each stallion was in contact with 9 to 15 mares, with rates of MHC-dissimilar mares ranging from 25–100% (no MHC-similar mare could be found for one of the stimulus stallions during the time he was available for the experiments). MHC sharing between mare and stimulus stallion was not significantly correlated to MHC sharing between mare and semen donor (Fisher exact test, n = 97, *P* = 0.16). Clinical cases of endometritis that necessitated treatment were observed during 13 cycles (13.4%) of in total 5 mares. Fresh semen analysis of the donor stallion over the duration of the experiments met acceptable standards (ejaculate volume [mean ± SD] = 22.4 ± 11.8 mL, sperm concentration 230.0 ± 76.8 × 10^6^ sperms/mL, sperm progressive motility = 80.4 ± 4.7%, total sperm count = 4.8 ± 2.3 × 10^9^ sperms).

**Figure 1:**
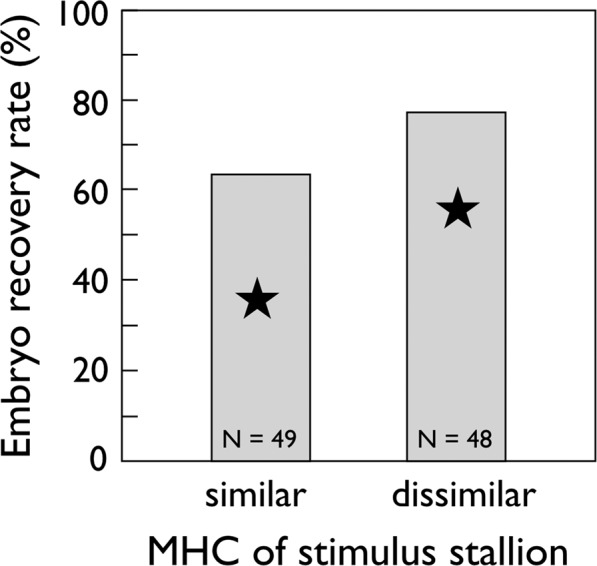
Embryo recovery rate in response to MHC social signaling. Total embryo recovery rate (%; grey boxes) in response to MHC sharing between mare and stimulus stallion. These rates were determined by embryo flushing 8 days after ovulation and are not significantly different (see text for power analysis and further statistics). The numbers of embryo flushing are given in the boxes. The stars provide the mean pregnancy rates observed in Burger *et al*.^9^ in an analogous experiment on other pair combinations but determined 14–17 days after ovulation.

In total 68 embryos could be collected from 67 flushings. Two embryos were collected in one case (the second embryo of this one case is ignored in the following statistics). One additional embryo could not be flushed but the mare was diagnosed pregnant after 13 days. The total 68 embryos correspond to an embryo survival rate of 70.1% which is higher than the average pregnancy rate of 45.7% observed in Burger *et al*.^9^ (χ^2^ = 22.5, n_1_ = 97, n_2_ = 191, *P* < 0.001). Embryo recovery rate was 77.1% when mares were in contact with an MHC-dissimilar stallion and 63.3% when they were exposed to an MHC-similar stallion (Table 1; Fig. 1). This difference was not significant (Table 1) but would correspond to an effect size of h = 0.30. While also MHC sharing to the semen donor did not seem to affect fertility (Supplementary Table S1), the occurrence of endometritis that required treatment was a significant negative predictor of embryo recovery rates (Table 1).

**Table 1:** Effects of sharing major histocompatibility complex (MHC) antigens with the stimulus stallion on presence or absence of embryos 8 days after ovulation.

model	effect tested	d.f.	logL	χ^2^	*P*
*MHC* + *endometritis* + *mare* + *stallion*		5	−54.8		
Endometritis + mare + stallion	MHC	4	−55.9	2.2	0.14
MHC + mare + stallion	endometritis	4	−58.1	6.5	**0.01**
MHC + endometritis + mare + stallion + MHC x endometritis	endometritis x MHC	6	−57.9	0	1.0
MHC + endometritis + stallion	mare	4	−54.8	0	1.0
MHC + endometritis + mare	stallion	4	−54.8	0.01	0.90
MHC + endometritis + mare + stallion + MHC x mare	MHC x mare	7	−54.8	0	1.0
MHC + endometritis + mare + stallion + MHC x stallion	MHC x stallion	7	−54.4	0.8	0.67

Likelihood ratio tests comparing generalized linear mixed models with MHC sharing (yes/no; “MHC”) and endometritis (yes/no) as fixed factors, and stimulus stallion (“stallion”) and mare identities (“mare”) as random factors. Reduced or amended models are compared to the reference model (italics). Significant *P*-values are emphasized in bold, d.f. = degrees of freedom, logL = log likelihood.

The quality of 61 of the 67 embryos were graded as “excellent”, 5 as “between excellent and good”, and one each as “poor” or “degenerated”^36^. MHC-sharing to the stimulus stallion had no effect on whether an embryo could be graded as “excellent” or not (Fisher exact test, n = 67, *P* = 0.70). Embryo diameter ranged from 213 to 1530 µm (mean = 764 µm) and was not significantly influenced by MHC-sharing to the stimulus stallion (linear mixed model, effect of MHC sharing: F_1,7.1_ = 2.2, *P* = 0.18). Endometritis was not more likely after exposure to MHC-similar than after exposure to MHC-dissimilar stimulus stallion, and stallion or mare identity seemed to play no role here (see non-significant interactions in Table 1).

All but 5 mares could be exposed to at least one MHC-similar and at least one MHC-dissimilar stallion, i.e., the full-factorial within-subject design was not fully complete. Excluding the mares that could only be exposed to either MHC-similar or –dissimilar stallions, and hence reducing the data set to a full-factorial within-subject design (24 mares, 88 embryo flushing), lead to similar outcomes (Supplementary Table S2).

Neither the rates of MHC sharing to stimulus stallion nor to the donor stallion were significantly different between the period when only frozen sperm could be used as compared to the period when only fresh sperm was used (Fisher exact test, n = 97, stimulus stallion: *P* = 0.12, donor stallion: *P* = 0.73). Using cryopreserved sperm in 2018 did not seem to affect the occurrence of endometritis (Fisher exact test, n = 97, *P* = 0.12) nor the rate of embryo recovery (Fisher exact test, n = 97, *P* = 0.08) as compared to using fresh semen in the years before. There were, however, non-significant tendencies of fewer cases of endometritis and higher rates of embryo recovery in 2018 when only cryopreserved sperm was used.

The rate of mares used in sport competitions was not significantly different between the present study and Burger *et al*.^9^ (Supplementary Table S3), but the mares of Burger *et al*.^9^ were more often used in sport competitions during the year the experiments took place than the mares that were used in the present study (Supplementary Table S3). The mares of the present study were also on average 2.5 years younger than the ones used in Burger *et al*.^9^ (Supplementary Fig. S1A). However, neither mare age at the time of the experiment nor being used in sport competitions significantly affected pregnancy rates as determined in Burger *et al*.^9^ (Supplementary Fig. S1B; Table S4).

## Discussion

Using an experimental protocol that is very similar to the one of Burger *et al*.^9^ allowed us (i) to estimate the expected effect size and plan our study accordingly, and (ii) to compare the outcome of the present study that focuses on the first 8 days of pregnancy with Burger *et al*.^9^ who included possible effects during embryo fixation in the endometrium 14–17 days after ovulation. From the effect size estimation, we had an >80% probability of finding a significant effect of the same size as Burger *et al*.^9^. This probability was higher than 80% because we used mostly within-subject comparisons that are statistically more powerful than the between subject comparisons that Burger *et al*.^9^ used. However, we did not find evidence for effects of MHC-linked social signaling on embryo recovery rates. We, therefore, conclude that the impact of MHC social signaling on fertility that Burger *et al*.^9^ found cannot, or at least not solely, be explained by maternal effects on embryo survival during the first 8 days of pregnancy.

Our observations do not allow to dismiss the possibility that MHC-social signaling affects embryo survival during the first 8 days because type II errors are difficult to exclude, and because we actually observed a statistically non-significant tendency towards higher embryo recovery rate after the mares had been exposed to MHC-dissimilar stallions than after they had been exposed to MHC-similar stallions. The corresponding effect size of h = 0.3 was smaller than the h = 0.40 that Burger *et al*.^9^ had observed. If the effect were real, we would need a minimum sample size of N = 166 to demonstrate it at α = 0.05 with a power of 80% in a balanced experimental design. This would correspond to nearly a doubling of the present sample size. However, the significant decline of embryo survival from day 8 (70.1%, present study) to days 14–17 (45.7%, Burger *et al*.^9^), and the decline of the observed effect sizes suggests that MHC-linked social signaling affects embryo survival at least during fixation in the endometrium.

The within-subject approach that we used to control for variance between mares is a valid option in horses because repeated embryo collections on the same mare do not affect embryo recovery rates^37^. By using only one semen donor stallion with adequate semen quality, we could minimize the potential variation in fertility due to the donor stallion. Importantly, the MHC similarity between the donor stallion and the mares was not correlated with the MHC similarity between the stimulus stallions and the mares.

The comparison between the present study and Burger *et al*.^9^ provides an useful estimate of the loss of embryos between day 8 and days 14–17 because we followed the experimental protocol of Burger *et al*.^9^ until the flushing of the embryos and even used the same stables. Our sample of mares was comparable to the one of Burger *et al*.^9^ with regard to the rates of Franches-Montagnes and the rates of mares used in sport competitions. However, our mares were studied over several cycles and were therefore less often used in sport competitions during the experimental year than the mares used in Burger *et al*.^9^. Because such use in sport competitions can sometimes affect fertility^38^, we tested for possible effects on fertility and found none. We also found no significant age effects on fertility. We therefore conclude that the observed differences in the timing of sport activities and in mean age were no factors that could be confounding in a comparison between the two studies. Occurrence of post-breeding endometritis could not be avoided in both studies but was only systematically recorded in the present study and could hence be integrated into the statistical analysis presented here. This further increased the statistical power relative to the sample size in the present study. It turned out that endometritis significantly affected embryo recovery rate but did not interact with MHC sharing and did therefore not influence our conclusions.

MHC-linked physiological and biochemical maternal decisions about acceptance or rejection of embryos could happen at several time points during gestation^13,39^. MHC-linked Bruce effects, i.e., a pregnancy block following exposure to an unfamiliar male^11^, typically happen during the pre-implantation period in the mouse (*Mus musculus*)^40^ but can be observed until mid to late pregnancy in the Prairie vole (*Microtus ochrogaster*)^41^. In horses, too, abortions induced by a change of the harem stallion could still be observed after several months of pregnancy^42^. Here we focus on another type of MHC-linked maternal decisions than can be seen as late form of inter-sexual selection^13^, and we focus on the oviductal passage of the embryo that takes more time in horses (144–156 h) than in many other species (e.g. pigs 48 h, sheep 66 h, mice 72 h, cows 72h^43^). Our results suggest that this type of MHC social signaling does not significantly influence embryo survival in the oviduct but mainly (or only) later during gestation. We also found no significant effects of MHC social signaling on embryo quality after 8 days.

Equine conceptuses move inside of the uterine lumen between day 8 and day 16 of gestation^29^. This seems important for maternal recognition of pregnancy, leading to maintenance of the corpus luteum and the synthesis of progesterone^25^, but the timing and the mechanisms of maternal recognition of pregnancy in the horse are still not sufficiently understood^44^. At that time, embryos have lost their zona pellucida and are surrounded by an acellular glycoprotein capsule that only disappears during implantation in the endometrium^45^. Several embryo-derived hormones and endometrium-derived proteins have been observed, but further studies on transcriptomes will help to better understand communication at this time stage^46^. We predict from our observations and the results of a previous study^9^ that this critical period of maternal recognition of pregnancy can be influenced by MHC-linked social signaling.

In conclusion, the present study in combination with a previous one^9^ allows to separate possible effects of MHC-linked social signaling on embryo survival during the first 8 days of the preimplantation period from possible effects after this first period when conceptuses move inside of the uterine lumen, i.e. between days 8 and 17 of gestation. Using a sample size and an experimental protocol that provides sufficient statistical power, we found no evidence for MHC-linked social effects on embryo survival during the first period. We conclude from comparing our embryo recovery rates with the pregnancy rates observed in Burger *et al*.^9^ that there is a significant loss of embryos in the period between days 8 and 17 of gestation. This suggests that maternal support of embryos during the period of fixation in the endometrium is important and can be biased by MHC-linked social signaling that happened around ovulation and fertilization.

## Materials and Methods

### Calculation of required sample size

We used the same experimental set-up that Burger *et al*.^9^ used when they found MHC social signaling to affect fertility in horses, except that we collected embryos already 8 days after ovulation by flushing the uterus. Their study could therefore to be used for the calculation of required sample size.

Power analysis was done in R 3.3.3^47^ with the pwr package^48^. The expected effect size h was calculated from Burger *et al*.^9^ who reported a mean pregnancy rate per stimulus stallion of p1 = 55.5% when mare and stimulus stallion were MHC dissimilar, and a mean pregnancy rate of p2 = 35.8% when mare and stimulus stallion were MHC similar. Using the formula

$${\rm{h}}=2\,\arcsin \,(\surd p1)-2\,\arcsin (\surd p2)$$

led to an observed effect size of h = 0.40. This effect size estimate was used to calculate the required minimum sample size for finding two proportions significantly different at α = 0.05 with a power of 80% (as suggested by Cohen^49^). The resulting minimal common sample size in the two groups was n_1_ = n_2_ = 49. In the present study, the corresponding numbers of reproductive cycles with embryo flushing were n_1_ = 49 and n_2_ = 48. However, because Burger *et al*.^9^ used a between-subject design, using the more powerful within-subject experimental design (as we did in order to control for some of the variance caused by differences among mares, see below) will increase the statistical power of finding an effect of h = 0.40 to >80%.

### Animals and infrastructures

The experiment took place at the Reproduction Center of the Swiss National Stud of Agroscope in Avenches, Switzerland, during four consecutive breeding seasons (March to September 2015–2018). Twenty-nine clinically healthy mares without foals (mean age at the beginning of the study 9.8 years, range 3–17 years) of various recognized Warmblood breeds (n = 26) and Franches-Montagnes (n = 3, a Warmblood-related local breed) were used for the experiments. This rate of Franches-Montagnes was not significantly different to the one in Burger *et al*.^9^ (Supplementary Table S3). Eight clinically healthy, sexually experienced stallions (mean age 13.9 years, range 6–21 years, all of Franches-Montagnes breed) were used as stimulus stallions. One further stallion (13 years old at the beginning of the study, Franches-Montagnes breed) was used as a semen donor. Four of the stimulus stallions were also used as stimulus stallions in Burger *et al*.^9^, but neither the sperm donor nor any of the mares that were used here had been used in this previous study. In order to further test for differences between the present sample of mares and the one of Burger *et al*.^9^, it was recorded for both studies whether and when the mares were used in sport competitions. All experimental animals had been vaccinated against influenza and dewormed one month before the start of the trials each year. Based on pedigree analyses and using the stud book of the Franches-Montagnes breed (Fédération Suisse du Franches-Montagnes, Studbook FM, Avenches, 2017), we found that mares and stallions had no common ancestors for at least 4 generations.

Two separate stables, each consisting of 8 boxes of 12 m^2^ separated by a corridor (4 boxes each side) were used for the experiments. One stallion per stable occupied a corner box and could walk freely in the corridor for 17 hours per day. The mares occupied up to seven of the other boxes during experimental runs (following the protocol of Burger *et al*.^9^ and using the same stables that they used in the Swiss National Stud). The other stallions were housed in separate stables without any contact to the mares. When not in the experimental stables, mares were kept in groups in open stables without any contact to the stallions.

### Experimental procedure

**Figure 2:**
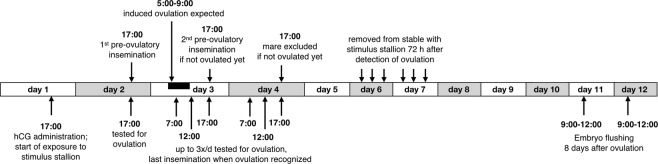
Timing of treatments and of the monitoring of ovulations. Exposure to the stimulus stallion started when a mare showed strong signs of estrous (see text for details) and hCG was injected to induce ovulation. During the following days the mare was up to 7 times tested for ovulation and up to three times instrumentally inseminated, depending on when ovulation happened. Exposure to the stimulus stallion ended 72 hours after detection of ovulation, and embryos were flushed 8 days after ovulation. Apart from the last step, this protocol is identical to the protocol of Burger *et al*.^9^.

Ovarian activity of the mares was regularly assessed by transrectal ultrasonography using a 7.5 MHz ultrasound with a 50 mm linear probe (MyLabOneVET, Esaote Spa, Florence, Italy). Figure 2 illustrates the timing of the treatments and the monitoring. Day 1 was defined as the day when transrectal ultrasonography revealed at least one follicle with a diameter of >35 mm, uterus edema at a minimum stage of 2^50^, and when the mare showed behavioral estrous signs after being shortly teased with a stallion (that was not further used in the experiment). At 17:00 on that day the mares were administered intravenously 1500 IU hCG (Chorulon, Intervet, Boxmeer, Netherlands) to induce ovulation that was expected to happen approximately 36–40 hours later^51^. Mares were assigned to one of the two experimental stables and exposed to the stimulus stallion in that stable.

At 17:00 on day 2, i.e. 24 h after hCG application (Fig. 2) mares were tested for ovulation. Ovulations were identified via ultrasonography by the presence of a normal corpus luteum and if no hemorrhagic follicles that would prevent the entrance of the oocyte into the oviduct^52^ were observed. Mares who had already ovulated at that time point were excluded from the experiment for that cycle. All others were instrumentally inseminated pre-ovulatory with semen from the donor stallion. On day 3 and if necessary day 4 (Fig. 2), mares were tested up to 3 times per day (at 7:00, 12:00, and 17:00) for ovulation. The mares were inseminated again directly after detection of ovulation with refrigerated semen (or frozen semen in 2018). When a mare had not ovulated 48 h after hCG application (i.e. 24 h after the first insemination), she was inseminated a second time pre-ovulatory and then a third time directly after the detection of ovulation. If the mare had not ovulated 72 h after hCG application (i.e., 24 h after the second insemination), she was excluded from the experiment for that cycle.

Transrectal ultrasonography was performed 24 h after detected ovulation in order to control for the presence of uterine fluid (post-breeding endometritis). If more than 20 mm of fluid was identified, mares received daily an intra-uterine lavage with warm physiological saline solution (NaCl 0.9%) and repeated systemic application of 20 IU oxytocin (Oxytocin, Stricker AG, Zollikofen, Switzerland) every 6 h during 24 h or, if uterine fluid persisted, for up to 72 h after ovulation. Mares stayed in contact with the stallion until 72 h after detection of ovulation. Total contact time between mare and stimulus stallion therefore ranged from 110 to 144 h, depending on the timing of ovulation. Embryo flushing took place between 9:00 and 12:00 of day 8 after detection of ovulation (Fig. 2).

### MHC determination

The MHC types of all experimental animals were defined serologically. Equine leukocyte antigens (ELA) class I and class II were determined via microcytotoxicity tests using alloantisera detecting 26 ELA-A (MHC class I) specificities, 5 MHC class II alleles, the ELA-C allele W21, and the allele W12 (unknown MHC class) according to Lazary *et al*.^53^ and as detailed in Jeannerat *et al*.^10^. The MHC types of the horses are shown in Supplementary Table S5. As in previous studies on horses^7–10^, a pair was classified as “MHC similar” if at least one ELA was shared between a stimulus stallion and a mare, and as “MHC dissimilar” if no ELA were shared. We decided against genotyping by MHC-linked microsatellites^54^ because microsatellites are more likely to over-estimate variation in MHC structural genes than serological methods^55^.

### Semen collection and preparation

During the reproductive seasons 2015–2017, fresh sperm was used from daily semen collections on a dummy using an artificial vagina (type Avenches). The gel fraction was filtered, and the volume of the ejaculate was measured and sperm concentration assessed with the Nucleocounter SP-100 system (ChemoMetec A/S, Allerød, Denmark). Semen was diluted with INRA 96 (IMV technologies, L’Aîgle, France) to a density of 100 × 10^6^ spermatozoa/mL, centrifuged at 600 g during 7 minutes in order to eliminate seminal plasma, and sperm were re-diluted in INRA 96 to obtain at least 3 insemination doses of a final quantity of 500 × 10^6^ spermatozoa in 10 mL volume each. A small portion of the semen was diluted to a density of 30 × 10^6^ spermatozoa/mL and analyzed for motility and velocity of sperm cells in 10 fields with a computer-assisted sperm analyzer (HTM-IVOS, version 12.1, Hamilton Thorne Biosciences, Beverly, USA). Ten mL of the diluted semen were used directly for insemination (pre-ovulatory) and the rest stored at 4 °C until used for the second and eventually further insemination(s) of the mare.

In winter 2017, the semen donor stallion had to be euthanized due to an acute injury (independent of the experiment), so that frozen semen collected in 2005 and 2006 was used for inseminating the mares in 2018. Neither the rates of MHC sharing to stimulus stallion nor to the donor stallion were significantly different between the periods when frozen or fresh sperm were used (Fisher exact test, n = 97, stimulus stallion: *P* = 0.12, donor stallion: *P* = 0.73).

Semen cryopreservation of the donor stallion was performed according to Weiss *et al*.^56^ and Janett *et al*.^57^. Briefly, after collection and removal of the gel fraction, semen was diluted with INRA 82-Hepes + 2% egg yolk to a density of 100 × 10^6^ spermatozoa/mL and centrifuged at room temperature at 600 g for 7 minutes. Thereafter, the supernatant was removed and the sperm pellet resuspended in a freezing extender (77% lactose solution 11%, 20% egg yolk, 3% glycerol) to a final concentration of 200 × 10^6^ spermatozoa/mL. After resuspension, semen was assimilated to a temperature of 4 °C for 30 minutes, packaged into 0.5 mL straws, and frozen in an automatic freezer (Minidigitcool 700 ZB 290, IMV Technologies, L’Aigle, France) at a cooling rate of 60 °C/min from +4 °C to −100 °C and then 30 °C/min from −100 °C to −140 °C. If ejaculates met acceptable standards after thawing (i.e., progressive motility of sperm ≥35%) the straws were stored in liquid nitrogen. For each insemination, four straws were thawed in a water bath at 37 °C for 30 s.

### Embryo collection and handling

Equine embryos are expected to enter the uterus around day 6 after ovulation^24^, but to avoid missing late arrivals, they were collected 8 days after ovulation by flushing the uterus following a standardized protocol routinely used in equine embryo transfer programs^58^. Embryo recover rate was used as approximation for embryo survival during the first 8 days. In preparation for non-surgical embryo flushing, mares were restrained in stocks and their vulva cleansed using iodine solution and water. Sterile physiological solution (lactated Ringer without glucose, Bichsel AG, Interlaken, Switzerland) at 38 °C was instilled into the uterus using a Foley catheter (IMV Technologies, L’Aigle, France), distributed into the lumen by gently moving the uterus transrectally, and re-collected through a Y-junction tubing into a 75 µm-filter (IMV Technologies, L’Aigle, France). Uterus flushing was continued by infusing and re-collecting repeatedly 1 to 3 liters of lactated Ringer until 5 liters had been used. The filter was searched for embryos under a stereomicroscope at 20x magnification by two persons who were naïve with respect to the MHC of the animals. If no embryo was present, the procedure was repeated with 5 additional liters of lactated Ringer. Twenty IU oxytocin (Oxytocin, Stricker AG, Zollikofen, Switzerland) were injected intravenously to provoke contraction and emptying of the uterus at the end of flushing. The quality of the embryos was assessed using the grading system described by McCue *et al*.^36^. In total 37 embryos, including 27 embryos of mares that had been exposed to both, an MHC-similar and an MHC-dissimilar stimulus stallion, were haphazardly picked to be sent to another laboratory for size measurements and further treatments in the context of another study (unpublished data). After embryo flushing, mares were intramuscularly injected 7.5 mg prostaglandin F_2α_ (Prosolvin, Virbac AG, Switzerland) to induce luteolysis. The next ovulation was not used for the experiment, but if another experimental run was possible with a given mare, it was done during the following spontaneous estrus. The mare was then assigned to another stimulus stallion. In three cases, the same mare x stimulus stallion combination was used again by accident.

### Data analysis

Statistical analyses were performed in R 3.3.3^47^ with the lme4 package^59^ and in Jmp 14.0.0 (www.jmp.com). Generalized linear mixed models were constructed on embryo recovery (yes/no) as dependent variable, MHC sharing to the stimulus stallion (yes/no), MHC sharing to the sperm donor (yes/no), and the occurrence of endometritis that required treatment (yes/no) as fixed factors, and the identity of the stimulus stallion and the mare as random factors. To test the significance of an effect, a model lacking or including an effect was compared to a reference model in likelihood ratio tests. A linear mixed model was used on embryo size as dependent variable, with MHC sharing to the stimulus stallion (yes/no) as fixed factor and mare identity and MHC sharing x mare identity as random factors. All *P*-values are two-tailed and considered significant if below 0.05.

### Ethical note

The experiments were approved by the local animal ethics committee (Etat de Vaud, Service Vétérinaire, permit numbers 2949a and 2950a) and performed in accordance with the relevant guidelines and regulations. No manipulations resulted in injuries. All animals had access to water ad libitum and were fed hay and cereals. Stallions were kept individually in boxes and were exercised daily during at least 1 h. Mares were turned out every day for 2 h on a paddock during the experiments and else kept in groups in open stables allowing free movement.

## Supplementary information

Supplementary information.

## Data Availability

The data used in this study are available from the Dryad Digital Repository (10.5061/dryad.s4mw6m936).

## References

[1] Davies, D. M. *The compatibility gene*. (Allen Lane, 2013).

[2] Neefjes J, Jongsma ML, Paul P, Bakke O (2011). Towards a systems understanding of MHC class I and MHC class II antigen presentation. Nat. Rev. Immunol..

[3] Ruff JS, Nelson AC, Kubinak JL, Potts WK (2012). MHC signaling during social communication. Adv. Exp. Med. Biol..

[4] Milinski M (2006). The major histocompatibility complex, sexual selection, and mate choice. Ann. R. Ecol. Evol. Syst..

[5] Wedekind C (2018). A predicted interaction between odour pleasantness and intensity provides evidence for major histocompatibility complex social signalling in women. Proc. R. Soc. B Biol. Sci..

[6] Havlicek J, Roberts SC (2009). MHC-correlated mate choice in humans: A review. Psychoneuroendocrinology.

[7] Burger D, Dolivo G, Marti E, Sieme H, Wedekind C (2015). Female major histocompatibility complex type affects male testosterone levels and sperm number in the horse (Equus caballus). Proc. R. Soc. B Biol. Sci..

[8] Burger D (2017). MHC-correlated preferences in diestrous female horses (Equus caballus). Theriogenology.

[9] Burger D (2017). Major histocompatibility complex-linked social signalling affects female fertility. Proc. R. Soc. B Biol. Sci..

[10] Jeannerat E (2018). Stallion semen quality depends on major histocompatibility complex matching to teaser mare. Mol. Ecol..

[11] Bruce HM (1959). Exteroceptive block to pregnancy in the mouse. Nature.

[12] Hausfater, G. & Hrdy, S. B. *Infanticide: comparative and evolutionary perspectives*. (Aldine, 2008).

[13] Wedekind C (1994). Mate choice and maternal selection for specific parasite resistances before, during and after fertilization. Phil. Trans. R. Soc. B.

[14] Yamazaki K (1983). Recognition of H-2 types in relation to the blocking of pregnancy in mice. Science.

[15] Yamazaki K (1986). Influence of a genetic difference confined to mutation of H-2k on the incidence of pregnancy block in mice. Proc. Natl. Acad. Sci. USA.

[16] Rülicke T, Guncz N, Wedekind C (2006). Early maternal investment in mice: no evidence for compatible-genes sexual selection despite hybrid vigor. J. Evol. Biol..

[17] Beydoun H, Saftlas AF (2005). Association of human leucocyte antigen sharing with recurrent spontaneous abortions. Tissue Antigens.

[18] Meuleman T (2015). HLA associations and HLA sharing in recurrent miscarriage: A systematic review and meta-analysis. Hum. Immunol..

[19] Ober C, Elias S, Kostyu DD, Hauck WW (1992). Decreased fecundability in Hutterite couples sharing HLA-DR. Am. J. Hum. Genet..

[20] Allen WR (2000). The physiology of early pregnancy in the mare. P. Annu. Conv. Am. Equin..

[21] Bézard J, Magistrini M, Duchamp G, Palmer E (1989). Chronology of equine fertilisation and embryonic development *in vivo* and *in vitro*. Equine Vet. J..

[22] Betteridge KJ, Eaglesome MD, Mitchell D, Flood PF, Beriault R (1982). Development of horse embryos up to twentytwo days after ovulation: observations on fresh specimens. Journal of Anatomy.

[23] Weber JA, Freeman DA, Vanderwall DK, Woods GL (1991). Prostaglandin E2 hastens oviductal transport of equine embryos. Biol. Reprod..

[24] Oguri N, Tsutsumi Y (1972). Non-surgical recovery of equine eggs, and an attempt at non-surgical egg transfer in horses. J. Reprod. Fertil..

[25] McDowell KJ, Sharp DC, Grubaugh W, Thatcher WW, Wilcox CJ (1988). Restricted conceptus mobility results in failure of pregnancy maintenance in mares. Biol. Reprod..

[26] Allen WR (2001). Fetomaternal interactions and influences during equine pregnancy. Reproduction.

[27] Stout TAE (2016). Embryo–maternal communication during the first 4 weeks of equine pregnancy. Theriogenology.

[28] Maillo V (2015). Oviduct-embryo interactions in cattle: two-way traffic or a one-way street?. Biol. Reprod..

[29] Ginther OJ (1983). Fixation and orientation of the early equine conceptus. Theriogenology.

[30] Ginther OJ (1998). Equine pregnancy: physical interactions between the uterus and conceptus. P. Annu. Conv. Am. Equin..

[31] Allen WR, Wilsher S (2009). A review of implantation and early placentation in the mare. Placenta.

[32] Palmer E, Driancourt MA (1980). Use of ultrasonic echography in equine gynecology. Theriogenology.

[33] McDonnell SM (2000). Reproductive behavior of stallions and mares: comparison of free-running and domestic in-hand breeding. Anim. Reprod. Sci..

[34] Burger D (2012). The potential effects of social interactions on reproductive efficiency of stallions. J. Equine Vet. Sci..

[35] Wedekind C (2002). Sexual selection and life-history decisions: implications for supportive breeding and the management of captive populations. Cons. Biol..

[36] McCue PM, DeLuca CA, Ferris RA, Wall JJ (2009). How to evaluate equine embryos. P. Annu. Conv. Am. Equin..

[37] Aurich C, König N, Budik S (2011). Effects of repeated embryo collection on embryo recovery rate in fertile mares. Reprod. Dom. Anim..

[38] Stout TAE (2003). Selection and management of the embryo transfer donor mare. Pferdeheilkunde.

[39] Eberhard, W. G. *Female control: sexual selection by cryptic female choice*. (Princeton University Press, 1996).

[40] Bruce HM (1961). Time relations in the pregnancy-block induced in mice by strange males. J. Reprod. Fertil..

[41] Stehn RA, Richmond ME (1975). Male-induced pregnancy termination in the prairie vole, *Microtus ochrogaster*. Science.

[42] Berger J (1983). Induced abortion and social factors in wild horses. Nature.

[43] Croxatto HB (2002). Physiology of gamete and embryo transport through the fallopian tube. Reprod. Biomed. Online.

[44] Klein C, Troedsson MHT (2011). Maternal recognition of pregnancy in the horse: a mystery still to be solved. Reproduction, Fertility and Development.

[45] Stout TAE, Meadows S, Allen WR (2005). Stage-specific formation of the equine blastocyst capsule is instrumental to hatching and to embryonic survival *in vivo*. Anim. Reprod. Sci..

[46] Klein C, Troedsson MHT (2011). Transcriptional profiling of equine conceptuses reveals new aspects of embryo-maternal communication in the horse. Biol. Reprod..

[47] R: A language and environment for statistical computing (R Foundation for Statistical Computing, Vienna, Austria, 2015).

[48] Champely, S. pwr: basic functions for power analysis. *R package version* 1.2–2 (2018).

[49] Cohen, J. *Statistical power analysis for the behavioral sciences*. (Lawrence Erlbaum Associates, 1988).

[50] Ginther, O. *Reproductive biology of the mare: basic and applied aspects*. Vol. 2nd edition (Equiservices, Inc., Wisconsin, 1992).

[51] Samper JC (2008). Induction of estrus and ovulation: why some mares respond and others do not. Theriogenology.

[52] Ginther OJ, Gastal EL, Gastal MO, Beg MA (2007). Incidence, endocrinology, vascularity, and morphology of hemorrhagic anovulatory follicles in mares. J. Equine Vet. Sci..

[53] Lazary S (1988). Joint report of the 5th international workshop on lymphocyte alloantigens of the horse, Baton Rouge, Louisiana, 31 October–1 November 1987. Anim. Genet..

[54] Tseng CT, Miller D, Cassano J, Bailey E, Antczak DF (2010). Identification of equine major histocompatibility complex haplotypes using polymorphic microsatellites. Anim. Genet..

[55] Miller D (2017). Polymorphism at expressed DQ and DR loci in five common equine MHC haplotypes. Immunogenetics.

[56] Weiss S, Janett F, Burger D, Hässig M, Thun R (2004). Einfluss der Zentrifugationsmethode auf die Qualität und Kryokonservierung von Hengstsamen. Schweiz. Arch. Tierh..

[57] Janett F (2006). Influence of repeated treadmill exercise on quality and freezability of stallion semen. Theriogenology.

[58] McKinnon AO, Squires EL (1988). Equine embryo transfer. Vet. Clin. North Am. Equine Pract..

[59] Bates, D., Maechler, M. & Bolker, B. lme4: Linear mixed-effects models using S4 classes. *R package version* 0.999375–39 (2011).

