# Supplementary material for: Embryo survival in the oviduct not significantly influenced by major histocompatibility complex social signaling in the horse
Source: Sci Rep. 2020 Jan 23;10:1056. doi: 10.1038/s41598-020-58056-w (PMC6978320; doi:10.1038/s41598-020-58056-w)
Supplement: Supplementary file 1 — Supplementary information. [file 41598_2020_58056_MOESM1_ESM.pdf]

Supplementary information

Embryo survival in the oviduct not significantly influenced by major histocompatibility complex social signaling in the horse

E. Jeannerat[1], E. Marti[2], S. Thomas[1], C. Herrera[3], H. Sieme[4], C. Wedekind[5#*], D. Burger[1#]

[1]Swiss Institute of Equine Medicine ISME, University of Berne, and Agroscope, Avenches, Switzerland

[2]Department of Clinical Research, Vetsuisse Faculty, University of Berne, Switzerland

[3]Clinic for Animal Reproduction Medicine, Vetsuisse Faculty, University of Zurich, Switzerland

[4]Unit for Reproductive Medicine – Clinic for Horses, University of Veterinary Medicine Hannover, Germany

[5]Department of Ecology and Evolution, Biophore, University of Lausanne, Switzerland

[#] shared senior authors

[#] shared senior authors

*correspondence: Claus Wedekind, claus.wedekind@unil.ch, orcid.org/0000-0001-6143-4716

Content

Supplementary Tables and Figure:

Table S1: Effects of MHC sharing with the sperm donor on the presence or absence of embryos 8 days after ovulation.

Table S2: Effects of MHC sharing on presence of embryo after 8 days tested only with mares that had been exposed to at least one MHC-similar and at least one MHC-dissimilar stallion.

Table S3: Comparing mares used in the present study with mares used in Burger et al.[1].

Table S4: Pregnancy rates of 185 mares used in Burger et al.[1] in relation to their use in sport competitions

Table S5: Serologically determined MHC antigens of the stallions and the mares.

Figure S1: Mare age versus success of insemination.

Supplementary Table S1. Effects of MHC sharing with the sperm donor on the presence or absence of embryos 8 days after ovulation. Likelihood ratio tests comparing GLMMs with MHC sharing (yes/no; "MHC_{donor}") and endometritis (yes/no) as fixed factors, and stimulus stallion ("stallion") and mare identities ("mare") as random factors. Reduced or amended models are compared to reference models (italics). Significant *P*-values are emphasized in bold, d.f. = degrees of freedom, logL = log likelihood.

model	effect tested	d.f.	logL	χ^2	*P*
MHC_{donor} + endometritis + mare + stallion		5	-55.0		
Endometritis + mare + stallion	MHC_{donor}	4	-55.9	1.8	0.18
MHC_{donor} + mare + stallion	endometritis	4	-58.5	7.1	**0.008**
MHC_{donor} + endometritis + mare + stallion + MHC_{donor} x endometritis	endometritis x MHC_{donor}	6	-58.0	0	1.0
MHC_{donor} + endometritis + stallion	mare	4	-55.0	0	1.0
MHC_{donor} + endometritis + mare	stallion	4	-55.0	0	1.0
MHC_{donor} + endometritis + mare + stallion + MHC_{donor} x mare	MHC_{donor} x mare	7	-55.0	0	1.0
MHC_{donor} + endometritis + mare + stallion + MHC_{donor} x stallion	MHC_{donor} x stallion	7	-55.0	0	1.0

Supplementary Table S3. Effects of MHC sharing on presence of embryo after 8 days tested only with mares that had been exposed to at least one MHC-similar and at least one MHC-dissimilar stallion (full-factorial within-subject design without missing cells, reducing the number of mares from 29 to 24 and the number of embryo flushing from 97 to 88). Likelihood ratio tests comparing GLMMs with MHC sharing (yes/no; "MHC") and endometritis (yes/no) as fixed factors, and stimulus stallion ("stallion") and mare identities ("mare") as random factors. Significant *P*-values are emphasized in bold, d.f. = degrees of freedom, logL = log likelihood.

model	effect tested	d.f.	logL	χ^2	*P*
MHC + endometritis + mare + stallion		5	-51.0		
Endometritis + mare + stallion	MHC	4	-51.7	1.4	0.23
MHC + mare + stallion	endometritis	4	-52.9	3.9	**0.05**
MHC + endometritis + mare + stallion + MHC x endometritis	endometritis x MHC	6	-53.1	0	1.0
MHC + endometritis + stallion	mare	4	-51.0	0	1.0
MHC + endometritis + mare	stallion	4	-51.0	0.2	0.69
MHC + endometritis + mare + stallion + MHC x mare	MHC x mare	7	-51.0	0	1.0
MHC + endometritis + mare + stallion + MHC x stallion	MHC x stallion	7	-50.3	1.8	0.54

Supplementary Table S3. Comparing mares used in the present study and in Burger et al.[1]. The table shows the rates of Franches-Montagnes mares used in both studies, and whether and when the mares had been used in sport competitions. Information about use in sport competitions was available for all mares of the present study (N = 29) and for 185 of the 191 mares used in Burger et al.[1].

Characteristics	Present sample	Burger et al. [1]	*P**
Franches-Montagnes breed	3 of 29 (10.3%)	18 of 191 (9.4%)	0.75
Used in sport competitions before	18 of 29 (62.1%)	122 of 185 (65.9%)	0.68
Used in sport competitions during year of experiment or the year before	6 of 29 (20.7%)	68 of 185 (36.8%)	0.10
Used in sport competitions during year of experiment	1 of 29 (3.4%)	37 of 185 (20.0%)	**0.03**

* Fisher exact test

Supplementary Table S4. Pregnancy rates of 185 mares used in Burger et al.[1] in relation to their use in sport competitions. Only pregnancies that resulted from the experimental treatment in Burger et al.[1] are taken into account here.

Characteristics	Not pregnant	Pregnant	*P**
Used in sport competitions before	78 of 112 (69.6%)	44 of 73 (60.3%)	0.21
Used in sport competitions during year of experiment or the year before	44 of 112 (39.3%)	24 of 73 (32.9%)	0.44
Used in sport competitions during year of experiment	24 of 112 (21.4%)	13 of 73 (17.8%)	0.58

* Fisher exact test

Supplementary Table S5. Serologically determined MHC antigens of the 8 stimulus stallions (A – H), 29 mares (1 - 29), and the semen donor stallion (i).

ID	MHC serotype
a) Stimulus stallions	
A	A2/A20 W22
B	A3/A5 W21 W23 W12
C	A2/Be108 W22
D	Be108 W21
E	A8/W11 BeIII W21 BeVIII
F	A2/Be108 W22
G	Be108 W21
H	Be108
b) Mares	
1	A3/A15 BeIII W21 W13
2	A15/A18 BeIII W21 W23
3	A15/W11 W13
4	A3/A15 BeIII W21 W13
5	A2/A5 W23
6	A6/A10 BeIII
7	A2/A19 W22 W23
8	A2/Be108
9	A2/A6 Be200
10	A2/A3 W21 W12
11	A2/A19 W23
12	A10/A19 BeIII W23 W13
13	A4/A15 BeIII W21
14	A2/A18 BeIII W22
15	W11 BeIII
16	A2/A15 BeIII W22
17	A3/A5 W21 W13 Be200
18	A4/A14 W21 W23
19	A1/A3 W21 W23 W13
20	A5/A6 W21 W23 Be200
21	A3 W21 W13
22	A3/A14 W12
23	A3/A15 W21 W12 BeIII
24	A2 BeVIII Be200
25	A5 W21 W13
26	A5/A9 W21 W23
27	A3 W21 W13 W12
28	A2/A5 W21 W23 W13
29	A2/A19 W22
c) Semen donor stallion	
i	A2/W11 BeIII

Supplementary Figure S1. Mare age versus success of insemination.

(A) Mare ages in Burger et al.[1] (N_1 = 188, the age of 3 mares used in this study was not known) and in the present study (N_2 = 29; t-test, t = 0.94, *P* = 0.01), and (B) success of insemination in relation to mare age at the time of the experiment in Burger et al.[1] (t = 0.94, *P* = 0.35). Tukey outlier boxplots with quartiles and whiskers; n.s. = not significant.

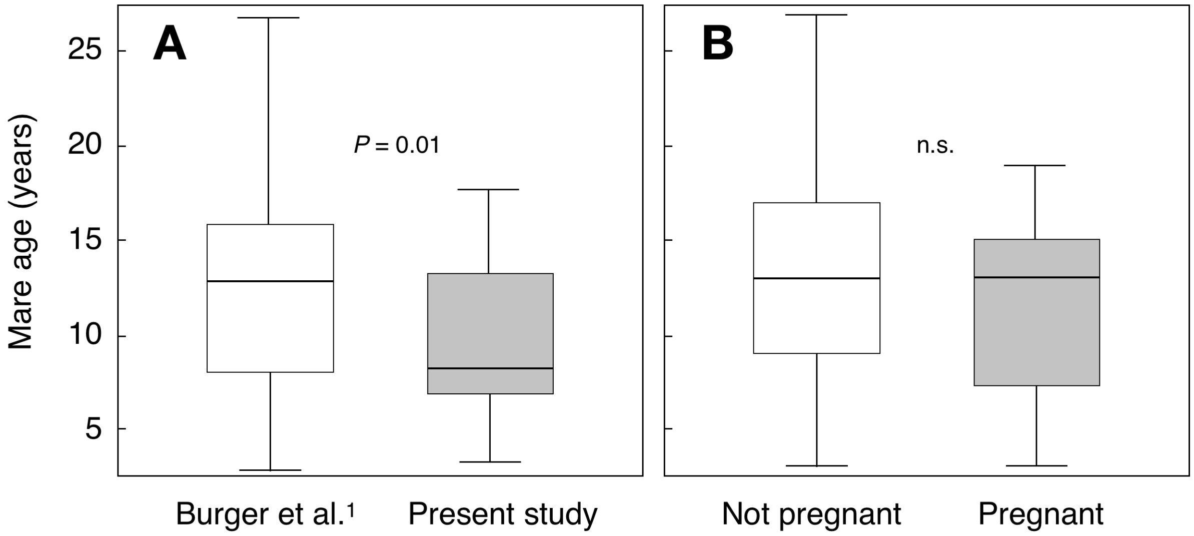

References cited

1 Burger, D. *et al.* Major histocompatibility complex-linked social signalling affects female fertility. *Proc. R. Soc. B Biol. Sci.* **284**, 20171824, doi:10.1098/rspb.2017.1824 (2017).
